# Auditory and visual novelty processing in normally-developing Kenyan children

**DOI:** 10.1016/j.clinph.2009.11.086

**Published:** 2010-04

**Authors:** Michael Kihara, Alexandra M. Hogan, Charles R. Newton, Harrun H. Garrashi, Brian R. Neville, Michelle de Haan

**Affiliations:** a The Centre of Geographical Medicine Research (Coast), Kenya Medical Research Institute (KEMRI), P.O. Box 428, Kilifi, Kenya; b Developmental Cognitive Neuroscience, Institute of Child Health, 30 Guilford Street, London, WC1N 1EH, UK; c Neurosciences Unit, Institute of Child Health, University College London, London, WC1N 3JH, UK; d Clinical Research Unit, London School of Hygiene and Tropical Medicine (LSHTM), London, UK

**Keywords:** Event-related potentials, Normative data, Novelty processing, Children, Africa

## Abstract

**Objective:**

The aim of this study was to describe the normative development of the electrophysiological response to auditory and visual novelty in children living in rural Kenya.

**Methods:**

We examined event-related potentials (ERPs) elicited by novel auditory and visual stimuli in 178 normally-developing children aged 4–12 years (86 boys, mean 6.7 years, SD 1.8 years and 92 girls, mean 6.6 years, SD 1.5 years) who were living in rural Kenya.

**Results:**

The latency of early components (auditory P1 and visual N170) decreased with age and their amplitudes also tended to decrease with age. The changes in longer-latency components (Auditory N2, P3a and visual Nc, P3a) were more modality-specific; the N2 amplitude to novel stimuli decreased with age and the auditory P3a increased in both latency and amplitude with age. The Nc amplitude decreased with age while visual P3a amplitude tended to increase, though not linearly.

**Conclusions:**

The changes in the timing and magnitude of early-latency ERPs likely reflect brain maturational processes. The age-related changes to auditory stimuli generally occurred later than those to visual stimuli suggesting that visual processing matures faster than auditory processing.

**Significance:**

ERPs may be used to assess children’s cognitive development in rural areas of Africa.

## 1 Introduction

Event-related potentials (ERPs) are used increasingly to assess basic sensory abilities that have important cognitive consequences in children (Burden et al., 2007; Byrne et al., 2001; Ceponiene et al., 2002a; Courchesne, 1978). They are not dependent upon language, are less likely to be influenced by culture than standard neuropsychological tests, and may be particularly useful in populations, such as those in Africa, that are currently without well-developed standardized psychological assessments. Few sensory evoked potential/ERP studies have been conducted with African populations (Elwan et al., 2003; Lombard, 2005; Mwanza et al., 2003; Oluwole et al., 2003), and most do not examine long-latency ERPs associated with cognition. Most studies focus on the effects of disease without establishing the development of brain potentials. The first step in the application of the ERP technique in detecting cognitive impairment in African children is the recording of normative ERP data in normally-developing African children. This is important, as it may be used to assess the nature and pace of neurocognitive development in these children. The aim of this study was to examine the development of ERPs associated with sensory and cognitive processing in children growing-up in rural Kenya.

One ERP response that has been well-studied across a wide age range is the response to deviance and/or novelty, because the child’s ability to detect and assimilate novel events is fundamental to cognitive development (Berg and Sternberg, 1985). Young infants’ ERP waveforms discriminate novel stimuli embedded in a train of frequent ‘familiar’ stimuli (Courchesne et al., 1981), and their ability to do so may influence their level of general intellectual functioning later in childhood (Lewis and Brooks-Gunn, 1981; Slater, 1997). In the ‘novelty oddball’ ERP task, three types of stimuli are presented: (i) one that is repeated at high probability (‘frequent’); (ii) another that is repeated at low probability (‘infrequent’); and (iii) a set of trial-unique novel stimuli presented at low probability. An advantage of this task over the classic 2-stimulus oddball task, which includes only a frequent and infrequent stimulus, is that it allows dissociation of response to low frequency versus novelty *per se* by comparing the two low frequency categories. A distinct waveform for the novel events is observed in auditory and visual modalities, and can be demonstrated even in passive tasks where the participant needs to look at or listen to the stimuli but does not need to actively respond (Courchesne, 1978; Picton, 1992; Polich and McIsaac, 1994; Squires et al., 1975). This characteristic of the ERP response to novel stimuli makes it particularly useful for studying cognitive development in young children, especially in those for whom language makes use of more traditional neuropsychological assessments difficult.

In children, the P1 (a positive peak around 100 ms after stimulus onset), the N2 (negative peak around 200 ms) and the P3a (a positive peak around 250–350 ms for novel stimuli) are the typical components observed in a passive auditory novelty oddball (Ceponiene et al., 1998, 2002b; Maatta et al., 2005). The P1 component is an obligatory cortical auditory evoked potential that reflects sensory encoding of auditory stimuli (Naatanen and Picton, 1987; Sharma et al., 1997). The auditory P1 has also been interpreted as an indicator of preferential attention to sensory inputs and is thought to reflect level of arousal (Key et al., 2005). The N2 is influenced by deviation in form or context of a prevailing stimulus (Naatanen and Picton, 1986), and is thought to be generated by diverse brain areas including the frontal and parietal cortical fields (Gomot et al., 2000), the superior temporal planes and Heschl’s gyrus (Takeshita et al., 2002). Studies using dipole source modelling suggest that the generators of the P1 mature slowly relative to the generators of the N2, possibly because of the slow development of superficial layers of the human auditory cortex (Ponton et al., 2000a). The P3a is interpreted as a neural correlate of the orienting response (Soltani and Knight, 2000), and has been associated with involuntary orienting of attention (Knight and Scabini, 1998). It may be elicited by behaviourally distracting/unexpected environmental sounds, e.g. telephone ring, dog bark or car horn, occurring among frequently repeated tones. The P3a component is attenuated in patients with lesions of the dorsolateral prefrontal cortex (Baudena et al., 1995; Friedman and Simpson, 1994; Knight, 1984), and the temporal lobe (Alho et al., 1998; Kotz et al., 2007), suggesting that this component is likely to be generated by a neural network involving the temporal and frontal lobes.

In the visual paradigm, we studied the face-sensitive N170. It is increasingly studied in children because of the important role of recognition and memory for faces in a child’s cognitive and social development (Grossmann and Johnson, 2007). This component is most prominent over the occipito-temporal region and in adults is maximal between 140 and 170 ms after stimuli onset (Bentin et al., 1996). It is thought to reflect the early perceptual encoding of the face, evidenced by its reduced amplitude when elicited by non-face compared to face stimuli (Bentin et al., 1996). The N170 is generated by regions including the fusiform gyrus (Shibata et al., 2002), the posterior inferior temporal gyrus (Bentin et al., 1996; Shibata et al., 2002), lateral occipito-temporal cortex (Bentin et al., 1996; Schweinberger et al., 2002) and the superior temporal sulcus (Henson et al., 2003; Itier and Taylor, 2004). An N170-like component is detectable from infancy, though this component continues to develop well into adolescence (de Haan et al., 2007). Larger amplitudes for faces compared to non-faces are observable from a young age, but the adult-like hemispheric distribution (whereby the component is larger over the right hemisphere) is not consistently seen until 12–13 years (Taylor et al., 2004).

The P3a component can be obtained by presenting infrequent distracter pictures in a series of frequent and infrequent familiar stimuli (Thomas and Nelson, 1996). This component, also called the novelty P3 by some authors, is maximal in the frontal/central scalp sites. It is also interpreted to reflect frontal lobe function (Friedman et al., 1993; Friedman and Simpson, 1994; Knight, 1984) resulting from an involuntary shift in attention (Courchesne, 1978; Escera et al., 1998). However, in children, a larger P3a response to visual novelty is not consistently reported (Thomas and Nelson, 1996; Van der Stelt et al., 1998). Instead children’s waveforms to novel visual stimuli typically display a frontally-distributed negative component (Nc) (Courchesne et al., 1975; Thomas and Nelson, 1996). The Nc component occurs between 400 and 800 ms and is the most recognizable and studied component in infant ERP research (Courchesne et al., 1981). It is elicited not only by novel stimuli, but also other salient, attention-getting stimuli such as the mother’s face (de Haan, 2007). It decreases with age over childhood and is not observed in adults (Courchesne et al., 1975). It has been suggested that the frontal P3a to visual novelty emerges as the Nc declines (Courchesne, 1978). The Nc is believed to be generated in frontal brain regions, a hypothesis supported by source analyses carried out on infant ERPs (Reynolds and Richards, 2005) and indirectly by parallels observed in the timing of developmental changes in Nc amplitude and the course of frontal cortical synaptogenesis (Courchesne, 1990; Shibasaki and Miyazaki, 1992).

In summary, there is a lack of information in African children on the development of commonly described ERP potentials. Such information is critical to the development of research into those social, environmental, and pathological influences on brain function to which children growing-up in Africa are frequently exposed. Thus we examined the development of two well-known ERP responses, those components elicited by stimulus novelty and the N170 component elicited by faces, in normally-developing children in rural Kenya.

## 2 Methods

The study was approved by the Kenya Medical Research Institute Ethical Review Committee. The study was conducted at the Centre of Geographical Medicine Research (Coast), which is situated in Kilifi, a coastal town in Kenya.

### 2.1 Subjects

A total of 178 children were identified from a community database. A fieldworker visited the homes of the selected children to give information about the study and obtain parental consent for their child to participate. Exclusion criteria included current prescription medication, and/or a history of neurological and/or developmental disorder, including delayed language and motor development compared to peers. This was further confirmed by the Ten Questions Questionnaire (Biritwum et al., 2001; Couper, 2002; Mung’ala-Odera et al., 2004) which was administered to all parents/guardians who consented to their child participating in the study, and included items addressing the child’s physical development, psychomotor skills, epilepsy and language development.

The sample included 86 boys (mean = 6.7 years, SD = 1.8 years) and 92 girls (mean = 6.6 years, SD = 1.5 years). Sixty-eight percent of the children were attending school (at least nursery school). Hearing was assessed using a Kamplex screening audiometer (PC Werth, London) and vision was assessed using a Sonksen–Silver chart (Salt et al., 1995). All children had normal vision and hearing.

### 2.2 Stimuli and recordings

This study involved both auditory and visual paradigms in which children listened to sounds or looked at pictures without responding overtly to them. Stimuli were presented using *Presentation*® software (Neurobehavioral Systems). Each child sat on an easy chair in a partially lit, sound-attenuated room facing a computer monitor placed approximately 70 cm away with two loud-speakers beside it.

#### 2.2.1 Auditory novelty paradigm

**Fig. 1:**
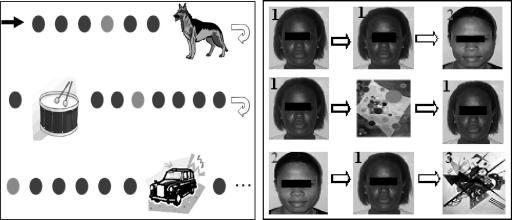
Visual representation of the auditory and visual paradigms. In the auditory experiment (left panel), the dark dot represents the frequent stimuli, the light dot, the infrequent stimuli and the pictures represent novel noises. The left panel consists of a frequently presented face (labelled 1), an infrequent face (2) and abstract paintings (3) used in the visual experiment.

The auditory paradigm was composed of three types of sounds: frequent and infrequent pure sinusoidal tones, and novel sounds. These tones and novel sounds were presented through two speakers placed in front of the children. Ten percent of the stimuli were infrequent tones (2 kHz, 200 ms long, 5 ms rise and fall time, 70 dB Sound Pressure Level, SPL), 10% were composed of novel noises e.g. dog bark, bell ring, etc. whereas the remainder were frequent tones of (1.5 kHz, 200 ms long, 5 ms rise and fall time, 70 dB SPL) (Fig. 1). The duration of the tones/noises was 200 milliseconds (ms) with a stimulus onset asynchrony of 700 ms. Two-blocks of 700 stimuli each were presented (560 frequent, 70 infrequent and 70 novels). Novel sounds were digitally adjusted in intensity so that they did not exceed 70 dB SPL as determined using a Bruel and Kjaer sound pressure meter. There were 14 different novel stimuli and were repeated a maximum of 5 times during the course of the experiment.

#### 2.2.2 Visual paradigm

The visual paradigm consisted of three types of images: an infrequently presented face and a frequently presented face (both were photographs of local people), and infrequently presented trial-unique, non-face abstract patterns (i.e. photographs of Kandinsky’s paintings) (Fig. 1). Stimuli were of equal size and presented at a visual angle of 16.78 × 14.25°. Two-blocks of 100 trials were presented in a random order, with 60% of the trials showing the frequent face, 20% infrequent face, and 20% non-face abstract picture stimuli (trial-unique). Participants were asked to look at a cross at the centre of the screen. The duration of the image presentation was 500 ms with an inter-stimulus interval of 3000 ms.

In total, the tasks took between 20 and 30 min to complete.

### 2.3 Measurements

#### 2.3.1 EEG recording

The EEG was recorded at a sampling rate of 500 Hz (band-pass 0.1–70 Hz) using SCAN (version 4.3, Neuroscan®, Compumedics, El Paso, Texas, USA; NuAMPs amplified), from 18 scalp electrodes (Ag/AgCl) placed according to the standard 10–20 system (Jasper, 1958). Data were recorded from midline leads at Fz, FCz, Cz and Pz, as well as lateral leads at Fpl, Fp2, F7, F8, T3, T4, T5, T6, P3, P4, O1, O2 and mastoid processes (A1, A2) in all children above 5 years of age. Horizontal and vertical electro-oculographs (HEOG and VEOG) were recorded by two electrodes on the outer canthus of the right eye and just below it respectively. All locations were referenced to a common Cz reference and subsequently re-referenced offline to averaged mastoids. For children 4–5 years old, we used nine scalp electrodes placed at Fz, FCz, Cz, Pz, A1, A2, HEOG, VEOG and Fpz, which acted as the ground electrode. Impedances were maintained at less or equal to 10 kΩ.

#### 2.3.2 ERP processing

EEG data were low-pass filtered offline at 20 Hz, baseline corrected, and waveforms were divided into epochs centred on stimulus presentation. An ocular artefact reduction algorithm on the Scan 4.3 software (Neuroscan Labs) was used to remove artefacts due to blinking. Any trials with amplitude deflections exceeding ±100 μV were rejected. A minimum of 20 trials for each stimulus was required for inclusion of an individual average ERP waveform.

### 2.4 Auditory ERP processing

Epochs −200 to 1000 ms were created offline centred on low and high tones and novel noises. The components of interest were the: P1, N2 and P3a, automatically detected in the time frames 70–110 ms, 210–270 ms and 270–370 ms, respectively, from midline locations (Fz, FCz, Cz and Pz) where these peaks are maximal (Fjell and Walhovd, 2004). The frequent stimuli immediately prior to each infrequent stimulus were selected for averaging to provide similar signal-to-noise ratios.

### 2.5 Visual ERP processing

Epochs −200 to 1500 ms were created offline centred on face and abstract picture stimuli. The components of interest were the face-specific N170, recorded as the most negative peak between 170 and 300 ms at T5 and T6, left and right temporal cortex respectively, and at midline; the P3a measured as the most positive peak between 350 and 650 ms and the negative component; and Nc, defined as the average amplitude between 400 and 850 ms (Latency data are not therefore provided for the Nc).

### 2.6 Data analysis and statistics

Data from six children in the auditory paradigm and four children in the visual paradigm were excluded due to excessive movement artefact. The peak amplitudes and latencies for the ERP components of interest from the remaining 172 (87 female and 85 male) and 174 (88 female and 86 male) children in the auditory and visual experiments, respectively, were explored across age categories: 4–5, 6–7, 8–9 and 10–12 year old bands. All analysis was conducted using SPSS for Windows, version 15 (SPSS Inc.®, Chicago, USA). Within-subject factors included site (X4: Fz, FCz, Cz and Pz) and stimuli (X3: frequent, infrequent and novel). The between-subject factors were age (X4: 4–5, 6–7, 8–9, 10–12) and sex (X2: male or female). The Greenhouse–Geisser correction is reported where applicable. We used the Tukey–Kramer test in the *post-hoc* analyses to correct for unequal sample sizes. Level of significance was set at *p* < 0.05.

## 3 Results

### 3.1 Auditory novelty oddball

**Fig. 2:**
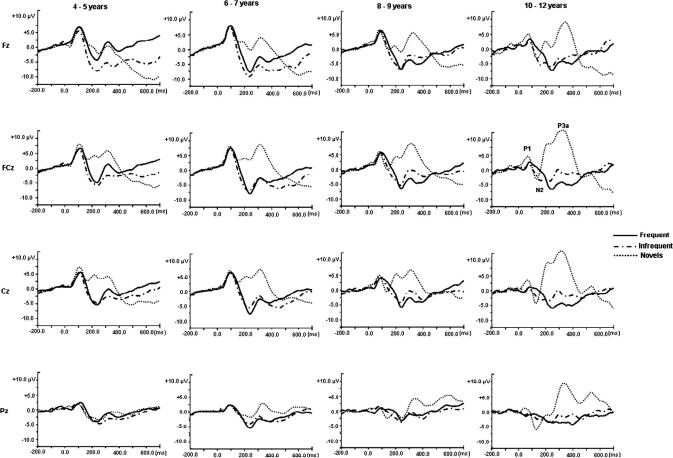
Grand averaged auditory ERP traces for frequent, infrequent and novel stimuli by age-group at midline scalp sites.

**Fig. 3:**
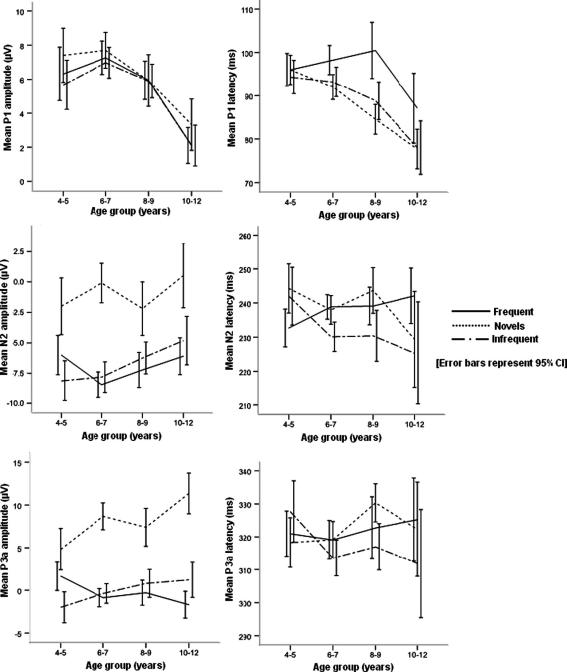
Normal development of auditory novelty processing in school-age children. Each line-graph shows plots of ERP components (latency and amplitude) averaged at midline electrodes (Fz, FCz, Cz, Pz) as a function of age-group.

Grand averaged waveforms in response to the frequent, infrequent and novel stimuli at midline electrodes for children in each age-group are in Fig. 2. The mean amplitudes and latencies for the different stimuli by age-group are provided in Table 1 (P1), Table 2 (N2), and Table 3 (P3a). Fig. 3 shows sub-diagraphs of the auditory components with age.

#### 3.1.1 P1 amplitude

There were significant main effects of Stimulus [*F*(2, 328) = 3.479, *p* = 0.032], Site [*F*(3, 492) = 80.655, *p* < 0.001] and Age [*F*(3, 164) = 3.459, *p* = 0.018]. These main effects were further explored in turn: P1 amplitudes associated with novel stimuli were larger than those associated with infrequent stimuli (novel > infrequent, *p* = 0.019), but not significantly different compared to frequent stimuli (*p* = 0.119); P1 amplitude was larger at fronto-central electrodes (i.e. Fz, FCz and Cz) compared to the posterior electrode (i.e. Pz) (Fz > Pz, *p* < 0.001; FCz > Pz, *p* < 0.001; Cz > Pz, *p* < 0.001); and, P1 amplitude decreased with age, but this reduction was only evident in older children, in particular, the mean P1 amplitude obtained from children aged 10–12 years-old was significantly lower than the mean P1 amplitude of the 4–5 year, 6–7 year and 8–9 year age-groups (*p* = 0.002, *p* < 0.001 and *p* < 0.001, respectively).

#### 3.1.2 P1 latency

There were significant effects of Stimulus [*F*(2, 328) = 4.424, p = 0.016] and Age [*F*(6, 164) = 5.236, *p* = 0.002]. The former reflected shorter latencies for novel and infrequent stimuli compared to frequent stimuli (*p* < 0.001 and *p* < 0.001, respectively). The main effect of age occurred due to decreasing P1 latencies with increasing age (significant age-group comparisons: 4–5 years > 8–9 years, *p* = 0.034; 4–5 years > 10–12 years, *p* < 0.001; 6–7 years > 10–12 years, *p* = 0.001; 8–9 years > 10–12 years, *p* = 0.005), irrespective of site and stimulus type. Significant interactions were found between Stimulus and Age [*F*(6, 328) = 3.047, *p* = 0.009], and Site and Age [*F*(9, 492) = 3.625, *p* = 0.004]. The interaction of Stimulus and Age occurred due to decrease in P1 latency associated with novelty with age (4–5 years > 8–9 years, *p* < 0.001; 6–7 years > 8–9 years, *p* = 0.001; 8–9 years > 10–12 years, *p* = 0.001), but this was not true of the mean latency associated with the frequent or infrequent stimuli which did not change significantly over age except for the 10–12 year olds whose latencies were shorter (Fig. 3). Similarly, the effect of Site by Age was explored. This interaction was driven by a decrease in the P1 latency with age at site Cz (4–5 years > 8–9 years, *p* = 0.003; 6–7 years > 8–9 years, *p* = 0.018; 8–9 years > 10–12 years, *p* = 0.049) but there was minimal difference at Pz (*p* = 0.259, *p* = 0.795 and *p* = 0.001 respectively).

#### 3.1.3 N2 amplitude

Analysis of N2 amplitude did not reveal any main effects, but there were interaction effects of Stimulus by Age [*F*(6, 328) = 2.731, *p* = 0.016], Stimulus by Site [*F*(6, 984) = 3.995, *p* = 0.003] and Stimulus by Site by Age [*F*(18, 984) = 2.656, *p* = 0.002]. The interaction of Stimulus and Age occurred because the magnitude of the N2 amplitude elicited by frequent stimuli was largest in children aged 6–7 years compared to 4–5 year-olds (*p* = 0.013) and 10–12 year-olds (*p* = 0.013) but not 8–9 year-olds (*p* = 0.175, *ns*). The interaction of Stimulus by Site occurs since the amplitude of the N2 elicited by the frequent stimuli is largest at Cz, whereas the N2 to infrequent stimuli was largest at Fz. Their resulting amplitudes are significantly different at Fz (frequent < infrequent, *p* = 0.024) and Cz (frequent > infrequent, *p* = 0.007) sites. The interaction of Stimulus by Site by Age occurred due to significantly smaller N2 amplitude elicited by the novel stimuli at Cz for children aged 10–12 years old compared to the other age-groups (10–12 years < 4–5 years, *p* = 0.004; 10–12 years < 6–7 years, *p* = 0.018; 10–12 years < 8–9 years, *p* = 0.001).

#### 3.1.4 N2 latency

There were significant interactions of Stimulus by Age [*F*(6, 328) = 2.731, *p* = 0.016], Stimulus by Site [*F*(6, 984) = 3.995, *p* = 0.003] and Stimulus by Site by Age [*F*(18, 984) = 2.656, *p* = 0.002]. The interaction of Stimulus by Age occurred because N2 latency associated with the infrequent stimulus was significantly longer for children aged 4–5 years compared to 6–7 years (*p* = 0.014) and 8–9 years (*p* = 0.040), but not those aged 10–12 years old (*p* = 0.351). The interaction of Stimulus by Site occurred because N2 latency associated with the frequent stimuli was significantly longer than that for the infrequent stimuli at fronto-central electrodes: Fz (infrequent < frequent, *p* = 0.022), FCz (infrequent < frequent, *p* = 0.003); Cz (infrequent < frequent, *p* = 0.020) but not at Pz (*p* = 0.861). This interaction was further modified by age as the N2 latency associated with infrequent stimuli in fronto-central electrodes was longer than that for frequent stimuli in children aged 4–5 years (infrequent > frequent, *p* < 0.026) but the reverse was true for the other age-groups: 6–7 years (infrequent < frequent, *p* < 0.001) and 8–9 year-olds (infrequent < frequent, *p* = 0.035), except 10–12 year-old which was not significant (*p* = 0.437).

#### 3.1.5 P3a amplitude

There were main effects of Stimulus [*F*(2, 328) = 40.983, *p* < 0.001] due to novel stimuli eliciting a larger P3a amplitude than frequent and infrequent stimuli (*p* < 0.001 and *p* < 0.001, respectively). A main effect of Site [*F*(3, 492) = 8.178, *p* = 0.001] occurred due to significantly larger P3a amplitudes in the fronto-central electrodes compared to posterior sites (FCz > Pz, *p* < 0.001 and Cz > Pz, *p* < 0.001). There was a significant interaction of Stimulus by Site [*F*(6, 984) = 8.411, *p* < 0.001] and Stimulus by Age [*F*(6, 328) = 4.300, *p* = 0.001]. The Stimulus by Site interaction occurred because the P3a amplitude associated with the novel stimuli was largest fronto-centrally compared to parietal electrodes for novel stimulus (FCz > Pz, *p* < 0.001; Cz > Pz, *p* < 0.001) but not for either frequent (*p* = 0.878 and *p* = 0.295) or infrequent stimuli (*p* = 0.350 and *p* = 0.385). The Stimulus by Age interaction was due to larger P3a amplitude associated with the novel stimuli in children aged 10–12 years old compared to all other age-groups (4–5 years < 10–12 years, *p* < 0.001; 6–7 years < 10–12 years, *p* = 0.005; 8–9 years < 10–12 years, *p* = 0.001). In general, P3a amplitude associated with novel stimuli increased with age – particularly between the age-groups 4–5 and 6–7 years, and 8–9 and 10–12 years (Fig. 3).

#### 3.1.6 P3a latency

There was a main effect of Site [*F*(3, 492) = 3.691, *p* = 0.025] due to significantly longer P3a latency at Pz electrode than fronto-central electrodes (Fz < Pz *p* = 0.009, FCz < Pz *p* = 0.005 and Cz < Pz *p* < 0.001). There was a significant interaction between Stimulus and Age [*F*(6, 328) = 2.263, *p* = 0.037], explained by prolonged P3a latency for novel stimuli in children aged 8–9 years compared to younger children [8–9 years > 4–5 years, *p* = 0.012; 8–9 years > 6–7 years, *p* = 0.006], but not compared to children aged 10–12 years [*p* = 0.757], although latencies were generally longer in this age-group compared to younger (4–5 and 6–7 years) children. There was also a significant interaction between Stimulus by Site [*F*(6, 984) = 2.556, *p* = 0.034], which occurred because the P3a component to frequent stimuli was of longer latency at Cz [*F*(2, 342) = 3.798, *p* = 0.033; frequent > novel, *p* = 0.022 and frequent > infrequent, *p* = 0.014] whilst at Pz, it was the response latency to novel stimuli that was prolonged [*F*(2, 342) = 9.674, *p* < 0.001; novel > frequent, *p* < 0.001 and novel > infrequent, *p* < 0.001].

### 3.2 Visual paradigm results

**Fig. 4:**
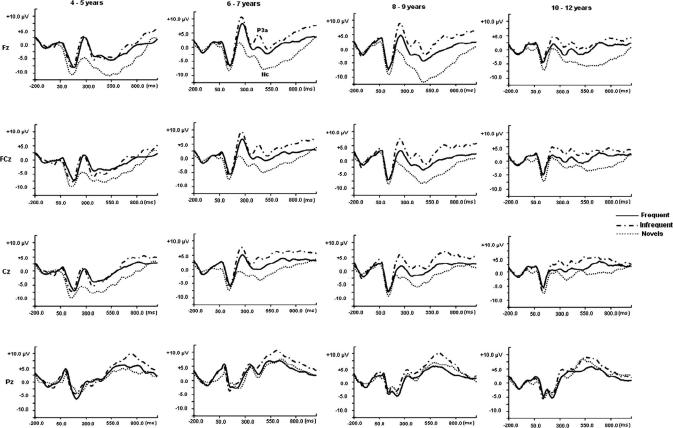
Grand average visual ERP traces for frequent, infrequent and novel stimuli by age-group at midline scalp sites. The visual P3a and Nc components. The N170 component is not shown here as it was derived from T5/T6 electrode.

In the visual paradigm, the components studied were the face-sensitive N170 at T5/T6 electrodes (but not examined in the youngest age-group due to the use of a slightly different EEG montage) and the P3a and Nc at midline electrodes (Fig. 4). The mean latencies and amplitudes associated with the visual stimuli over age and by stimulus are provided in Tables 4 and 5.

#### 3.2.1 N170 amplitude

**Fig. 5:**
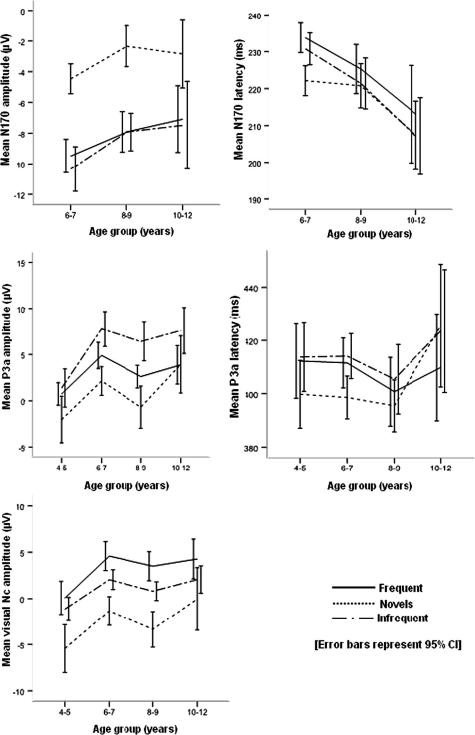
Normal development of visual novelty processing in school-age children. Line-graphs for N170 show ERP components (latency and amplitude) averaged at T5 and T6, while P3a and Nc are averaged at Midlines (Fz, FCz, Cz, Pz) as a function of age-group. Data for 4–5 year olds not available for the N170.

There was a significant main effect of Stimulus [*F*(2, 254) = 18.961, *p* < 0.001] which occurred due to a significantly smaller magnitude of the N170 amplitude associated with novel non-face stimuli compared to the N170 amplitudes associated with frequent and infrequent faces (*p* < 0.001 and *p* < 0.001, respectively); a finding that may be expected considering the role of the N170 in face processing. There was also a main effect of Age [*F*(2, 127) = 4.818, *p* = 0.010], explained by the N170 decreasing in magnitude with age (6–7 years > 8–9 years, *p* = 0.022; 6–7 > 10–12 years, *p* = 0.042); though the difference between 8–9 and 10–12 year-old children was not significant (*p* = 0.611) (Fig. 5). There was no significant main effect of site [*F*(1, 127) = 0.000, *p* = 0.998] indicating a lack of hemispheric difference.

#### 3.2.2 N170 latency

Analyses of the N170 latency revealed a main effect of Stimulus [*F*(2, 254) = 7.486, *p* = 0.001] due to significantly shorter N170 latency associated with novel non-face stimuli compared to N170 latencies elicited frequent and infrequent faces (novel < infrequent, *p* = 0.001 and novel < frequent, *p* < 0.001). There was a trend towards decreasing N170 latencies with age but the difference did not reach significance (*p* = 0.055). There were interaction effects of Stimulus by Age [*F*(4, 254) = 2.975, *p* = 0.022] and Stimulus by Site [*F*(4, 254) = 3.441, *p* = 0.037]. The interaction of Stimulus and Age occurred due to decrease in N170 latency associated with infrequent stimuli with increasing age (6–7 years > 8–9 years, *p* = 0.024; 8–9 years > 10–12 years, *p* = 0.024): this was irrespective of laterality. The interaction of Stimulus by Site occurred due to longer N170 latency associated with the frequent stimuli compared to the infrequent stimuli at right hemisphere – T6 (frequent > infrequent, *p* = 0.012) but not left hemisphere – T5 (*p* = 0.054).

#### 3.2.3 P3a amplitude

There were main effects of Stimulus [*F*(2, 332) = 9.512, *p* < 0.001], Age [*F*(3, 166) = 7.333, *p* < 0.001] and Site [*F*(3, 498) = 25.031, *p* < 0.001]. P3a amplitude associated with the infrequent stimuli was larger compared to P3a amplitude elicited by the frequent and novel stimuli (*p* < 0.001 and *p* < 0.001, respectively), and amplitude associated with frequent stimuli was larger than that of novel stimuli (*p* < 0.001). Children aged 4–5 years had significantly smaller amplitudes in general than other age-groups (4–5 years < 6–7 years, *p* = 0.004; 4–5 years < 8–9 years, *p* = 0.032 and 4–5 years < 10–12 years, *p* = 0.030) but the trend was not linear with increasing age (e.g. 6–7 years > 8–9 years, *p* = 0.036). The main effect of site occurred due to larger P3a amplitudes at Pz compared to other sites (Fz < Pz, *p* < 0.001; FCz < Pz, *p* < 0.001; Cz < Pz, *p* < 0.001). There was also an interaction of Stimulus by Site [*F*(6, 960) = 10.678, *p* = 0.001]. The interaction was due to significantly larger P3a amplitude associated with frequent stimuli compared to novel stimuli at fronto-central electrodes (Fz: frequent > novel, *p* < 0.001; FCz: frequent > novel, *p* < 0.001; Cz: frequent > novel, *p* < 0.001) but not at posterior sites (Pz: *p* = 0.447).

#### 3.2.4 P3a Latency

There were main effects of Stimulus [*F*(2, 332) = 6.099, *p* = 0.003] and Site [*F*(3, 498) = 4.036, *p* = 0.022]. The main effect of stimulus occurred due to significantly shorter latency associated with novel stimuli compared to P3a latencies associated with both frequent and infrequent stimuli (*p* = 0.009 and *p* < 0.001, respectively). The main effect of site was due to shorter P3a latencies at fronto-central sites compared to Pz (Fz < Pz, *p* = 0.009; FCz < Pz, *p* = 0.005 and Cz < Pz, *p* < 0.001). There was also an interaction of Stimulus by Site [*F*(6, 960) = 4.315, *p* = 0.002]. An examination of this interaction revealed significantly shorter P3a latency associated with the novel stimulus in frontal-central sites (Fz: novel < frequent, *p* < 0.001 and novel < infrequent, *p* < 0.001; FCz: novel < frequent, *p* = 0.001 and novel < infrequent, *p* < 0.001; Cz: novel < infrequent, *p* = 0.028, although novel < frequent, *p* = 0.070), but the difference was not significant at Pz (*p* = 0.826 and *p* = 0.648, respectively).

#### 3.2.5 Nc component averaged amplitude

There were main effects of Stimulus [*F*(2, 232) = 24.218, *p* < 0.001], Site [*F*(3, 498) = 65.944, *p* < 0.001] and Age [*F*(3, 166) = 6.166, *p* = 0.001]. These main effects were explored in turn. The effect of stimulus occurred since the magnitude of the Nc elicited by the novel stimuli was larger than that elicited by frequent or infrequent stimuli (*p* < 0.001 and *p* < 0.001 respectively). The frequent stimulus elicited a larger Nc compared to the infrequent stimulus (*p* < 0.001). The main effect of site was due to a decrease in the magnitude of the Nc from anterior to posterior brain sites (Fz > FCz, *p* < 0.001; FCz > Cz, *p* < 0.001; Cz > Pz, *p* < 0.001) (Fig. 5). The main effect of age occurred due to larger Nc in children aged 4–5 years than other age-groups (4–5 years > 6–7 years, *p* < 0.001; 4–5 years > 8–9 years, *p* = 0.011; 4–5 years > 8–9 years, *p* = 0.027). There was an interaction Stimulus by Site [*F*(6, 996) = 11.208, *p* < 0.001]. This interaction occurred because the Nc component associated with the novel stimuli was larger than that associated with the frequent stimuli at fronto-central sites (Fz: novel > frequent, *p* < 0.001; FCz: novel > frequent, *p* < 0.001; Cz: novel > frequent, *p* < 0.001) but not at Pz (*p* = 0.933).

## 4 Discussion

This study describes the development of novelty processing of auditory and visual ERPs in children aged 4–12 years living in a rural Kenya. The components elicited in our tasks have been described in previous studies of school-age children that used similar experimental conditions (Albrecht et al., 2000; Ceponiene et al., 1998, 2002c; Ponton et al., 2000a; Taylor et al., 1999) and are thought to represent perceptual–cognitive mechanisms. However, such comprehensive description of normal ERP development has not been reported in children living in Africa. We discuss two main themes in our data: age and stimulus effects, before examining the profile of each of the auditory and visual ERP components in greater detail.

A number of our findings indicate change in the timing and magnitude of ERP components across our age range. In summary, the latency of early components (auditory P1 and visual N170) decreased with age with their amplitudes also tending to decrease with age, while the changes occurring in longer-latency components were more modality-specific. More specifically, the later-occurring auditory P3a showed the opposite pattern to that seen with early components: i.e. an increase in both latency and amplitude with increasing age, whilst Nc amplitude decreased with age, the amplitude of the visual P3a showed nonlinear changes with age, and the latency of the visual P3a did not change with age. This pattern of development change in amplitudes and latencies was common across all stimulus types within each modality, and thus likely represents changes in the general processing of auditory and visual information in the brain. In general, a decrease in component latency with increasing age is consistent with data obtained from non-African children of similar age range (Ceponiene et al., 2002b; Ponton et al., 2000b). Amplitudes also had similar age-effects as those reported in previous studies, with the auditory P1 decreasing with age (Ceponiene et al., 2002b; Ponton et al., 2000b; Sharma et al., 2002). These findings are likely to represent brain maturational processes (Hogan et al., 2005; Ponton et al., 2000a), particularly white matter development (Barnea-Goraly et al., 2005; Eggermont, 1988).

Stimulus effects were present in even the youngest children, and evident in early (e.g. auditory P1 and visual N170) and late (e.g. auditory P3a and visual Nc) components. Specifically, the auditory P1 and P3a components differentiated stimulus novelty, controlling for stimulus frequency, irrespective of age, with novel stimuli eliciting components of greatest magnitude. Otherwise stated, there is a greater brain response to novel unexpected sounds than to sounds that simply occur with less frequency (infrequent stimuli). Conversely, the P3a in the visual paradigm was largest to the infrequently presented face, perhaps reflecting an inherent bias towards face processing, and in particular the allocation of attention to those faces that are still being learnt, over abstract pictures that are not as socially important. The visual Nc was largest for novel stimuli, and thus more responsive to the picture stimuli than the face stimuli. This component is typically found in very young children, which is consistent with a rapid decline in its amplitude with increasing age in our study. Components associated with face processing continue to develop into adolescence (de Haan et al., 2007), perhaps reflected in the decrease in the strength of the Nc elicited by novel abstract pictures over faces, and the consistent P3a preference for infrequent faces over novel abstract pictures in our data.

Importantly, our study also found that the way particular stimuli were processed changed with age, as revealed by Stimulus by Age interactions. For auditory stimuli, the P3a to novel stimuli, but not infrequent or frequent stimuli, increased in amplitude and latency with age. Also for the novel stimuli only, the auditory P1 latency decrease with age. The N2 to novelty did not change by age, but the amplitude and latency of the N2 did change for frequent and infrequent stimuli by age: (a) the N2 amplitude for frequent stimuli tended to be largest in the middle age-groups over 6–9 years and (b) the latency of the N2 over fronto-central sites was greater for infrequent than frequent stimuli in the youngest group, but showed the opposite pattern in older children. For visual stimuli, the Stimulus and Age variables tended to show main effects, indicating that the way novelty was processed changed less with age for visual than auditory stimuli. One exception to the lack of Stimulus by Age interactions for the visual task was that the latency of the N170 to infrequent stimuli increased with age.

### 4.1 Processing of auditory novelty

The P1 component is interpreted as a marker of preferential attention and is thought to reflect the level of arousal (Key et al., 2005). In this study, we showed a decrease in auditory P1 amplitude with increasing age between 7 and 12 years. This finding has also been obtained in other studies with short inter-stimulus intervals (Ceponiene et al., 2002b; Sharma et al., 2002). The P1 component, which is an obligatory component, is said to be an objective measure of cortical auditory function in children (Naatanen and Picton, 1987; Sharma et al., 1997). The decrease in P1 latencies and amplitudes can be viewed as maturation of the auditory evoked potentials (Ponton et al., 2000a). The continued development of the amplitude of the P1, when no similar effects were observed for N2, is consistent with the view that the generators of the P1 take longer to mature than those of the N2 (Ponton et al., 2002). The decrease in the amplitude and latency of P1 is also said to reflect the decline in synaptic density and increased intracortical myelination (Ceponiene et al., 2002b) and maturation of auditory pathways (Sharma et al., 2002). An alternate explanation for these findings could be due to an overlap of the emerging N1 component which is usually absent in younger children (Ceponiene et al., 2002b) causing the amplitude to diminish and the latency to decrease.

The auditory N2 is thought to originate bilaterally in the auditory cortex of the superior temporal lobes (Gomot et al., 2000). It reflects attention orienting and its decrease with age may reflect inhibitory attention control (Satterfield et al., 1994). In the present study, the N2 latency did not show age-related novelty effects but the amplitude decreased with age, a finding shown in a previous study using a similar inter-stimulus interval (Ceponiene et al., 2002b). The N2 latency has been reported to decrease with age (Ceponiene et al., 2002c; Enoki et al., 1993; Fuchigami et al., 1993; Goodin et al., 1978), remain unchanged (Cunningham et al., 2000) or even increase with age (Ponton et al., 2000b) between 5 and 15 year-old children. The differences in our results could have been due to much shorter ISI (700 ms) since all the other studies have used ISI longer than 1 s. Ceponiene and her colleagues (2002) used a similar short ISI and their findings did not show age-effects for children between 4 and 9 years. The decrease in N2 amplitude is thought to be a result of inhibitory control as the children develop (Ceponiene et al., 2002b).

The P3a component is assumed to reflect an involuntary attention switch from the actual focus of voluntary attention to the eliciting sound (Escera et al., 1998; Knight and Scabini, 1998), and is generated in the frontal lobes (Friedman et al., 1993; Friedman and Simpson, 1994; Knight, 1984). In our findings, novel auditory stimuli elicited a P3a, maximal over the fronto-central region. This finding has also been described by previous authors (Courchesne et al., 1984; Escera et al., 2000; Knight, 1984; Polich, 2007). In the present study, the P3a latencies associated with novel stimuli tended to increase with age though only the 8–9 year-old children had significantly longer latencies compared to the other age-groups. Most studies have reported a decrease in P3a latencies with age (Courchesne, 1978; Cycowicz et al., 1996; Fuchigami et al., 1995; Gumenyuk et al., 2001, 2004) between 5 and 16 years. An explanation for our different findings may be our difficulty to distinguish between the early and the late P3a (lP3a). The detection of the greatest positive peak within the given time range could have resulted in the selection of the early P3a in some children, and the late P3a in others. In adults, the lP3a is maximal frontally and does not invert polarity over the posterior sites (Escera et al., 1998) and has a peak latency of 250–350 ms while the eP3a peaks much earlier at 200–250 ms. A few children did not have double peaks and the maximal peak between 270 and 370 ms was taken to be the lP3a. A recent study found no significant difference in the latency of the P3a by age having used comparable age-groups of children aged between 5 and 12 years and having a similar non-response paradigm as the present study (Brinkman and Stauder, 2008). Another study’s preliminary results showed that the P3a’s of children were unclear and inconsistent (Segalowitz and Davies, 2004). In the present study, the mean amplitude of the P3a of older children were significantly larger compared to other age-groups. This component has also been shown to increase from 5 to 12 years (Ferri et al., 2003) or to remain unchanged within similar age ranges (Brinkman and Stauder, 2008).

Overall, our results suggest that the way children process auditory novelty changes with age. Quicker P1’s, smaller N2’s and larger amplitude P3a’s could reflect a faster orienting to novelty with age (P1) combined with better inhibitory control (N2).

### 4.2 Processing of visual novelty

In visual paradigms, the inclusion of trial-unique “novel” stimuli in a series of frequent and infrequent stimuli typically produces a P3a in adults that is maximal at the fronto-central electrodes (Thomas and Nelson, 1996). Our findings however revealed a P3a that was larger to infrequent stimuli compared to novel stimulus at all midline electrodes. Comerchero and Polich (1999) have demonstrated that the degree of infrequent/frequent stimuli discrimination difficulty determines the P3a generation (Comerchero and Polich, 1999). They argued that when the discrimination was easy, the P3a amplitude was larger for the infrequent stimuli than the distractor (novel) but when it was difficult, it was the distractor that had a larger amplitude (Comerchero and Polich, 1999). Based on the present results, one may speculate that the distinct difference between the frequent and infrequent face in the context of highly abstract novel stimuli resulted in a larger P3a to infrequent than novel stimuli. Its latency however revealed no age-effects while the amplitude showed a nonlinear change with age. A recent study with comparable age-group and using a 3-stimulus visual paradigm found age-related effects for P3a amplitude in children but not the latency (Stige et al., 2007). Their results indicated a main effect of age on the P3a amplitude due to significant decrease with age. However, there are very few studies reporting visual P3a in children and there is need for more investigation of its development in children and in relation to the Nc.

The Nc is thought to reflect enhanced attention to surprising, interesting, or important stimuli such as novel stimuli (Courchesne, 1978; de Haan et al., 2003). In the present study, the amplitude of the Nc component was larger in younger than older children, a finding consistent with prior research showing a decline in the Nc with age (Courchesne, 1978). The Nc had a fronto-central maximum and was larger for novel than frequent or infrequent stimuli, results also consistent with prior studies (Ackles and Cook, 1998; Courchesne et al., 1981; Stauder et al., 2006).

### 4.3 Face processing

The N170 was more positive to non-face (novel) stimuli then to face (frequent or infrequent) stimuli at all ages, a finding consistent with prior studies showing that the N170 is larger to faces then non-faces from the age of 4–5 years (Taylor et al., 2004). The latency of the N170 to infrequent stimuli decreased and its amplitude became more positive with age. These findings are similar to the report by Taylor et al. (2004), who found N170 latencies decreased linearly with age while N170 amplitudes became more positive from age 4 to 11 years and then became more negative after age 11 years. We did not observe a hemispheric difference in N170 amplitude, a result also consistent with prior studies suggesting that a consistent hemispheric difference in favour of the right side does not emerge until 12–13 years (Taylor et al., 2004).

The profile of processing auditory and visual events changed with age. The age-related changes to auditory stimuli generally occurred later than those to visual stimuli suggesting that visual processing matures faster than auditory processing. Processing of novelty also showed age-related changes in the auditory but not the visual task. Overall, our results provide normative data to the novelty ERP paradigm of normally-developing African children against which patient populations can be compared to determine developmental differences. We observed age-related effects both in latencies and amplitudes of components suggesting that the manner in which the brain engages in processing the various stimuli differs with age. Moreover, there may be subtle differences in the ERP activity between children in the West and those in Africa that could influence the interpretation of data, e.g. an increase in novelty P3a latency through late childhood. We found that ERP paradigms were tolerated well by Kenyan children and thus can be used to study the effects of cerebral insults and provide an alternative methodology of assessing perceptual–cognitive development in patient groups for whom more typical standardised neuropsychological assessments are unavailable. Our paradigms focused on components related to diverse brain areas, including prefrontal regions (P3a; Nc), ventral occipito-temporal pathways (N170) and superior temporal regions (N2), and thus may be useful in targeting which brain regions are most influenced by different disease processes. Whilst large data sets may be needed to provide a robust normative framework for clinical assessment and treatment prediction, we demonstrate the feasibility of collecting ERP data from large numbers of African children, and document similarities and subtle differences in the general developmental profile with those documented in Western populations.

## Figures and Tables

**Table 1:** Means and standard deviations for auditory P1 latencies (ms) and amplitudes (μV) at midline sites by age-group (years).

		4–5 years				6–7 years				8–9 years				10–12 years			
		Amplitude		Latency		Amplitude		Latency		Amplitude		Latency		Amplitude		Latency	
		Mean	SD	Mean	SD	Mean	SD	Mean	SD	Mean	SD	Mean	SD	Mean	SD	Mean	SD
Fz	Infrequent	6.29	4.7	95.6	13.6	8.54	4.7	92.0	16.3	7.32	3.4	87.3	13.9	3.48	2.7	78.6	10.1
	Novel	8.13	6.9	96.5	12.2	9.34	5.3	91.4	14.1	7.50	6.0	84.3	12.0	5.93	4.2	77.4	6.9
	Frequent	7.77	5.3	97.4	12.9	9.22	5.2	97.8	13.7	7.49	4.6	99.0	22.4	3.82	3.2	86.2	15.1

FCz	Infrequent	7.10	5.6	96.5	14.0	8.40	4.6	93.4	17.2	7.14	3.7	87.8	14.8	2.85	2.5	78.4	10.2
	Novel	9.63	6.0	99.3	11.7	9.25	5.5	92.8	13.2	7.33	6.0	83.6	11.4	5.29	3.4	76.8	6.8
	Frequent	7.57	5.6	96.8	13.5	8.71	5.2	99.1	15.5	7.60	4.4	102.7	22.7	3.01	2.6	86.2	15.6

Cz	Infrequent	6.20	5.5	95.7	14.0	7.30	4.6	94.3	18.0	6.06	4.0	88.2	18.1	1.82	2.5	85.0	17.4
	Novel	8.62	5.2	98.9	12.1	8.26	5.6	92.8	15.3	6.19	5.5	83.1	12.0	4.05	3.5	76.2	6.5
	Frequent	6.38	5.3	97.3	13.9	7.56	4.7	99.1	16.5	6.20	3.9	100.0	24.7	2.20	1.9	86.6	16.1

Pz	Infrequent	3.10	3.7	89.6	15.8	3.65	4.1	93.1	20.6	3.04	3.7	92.1	20.4	−0.10	2.2	80.4	15.3
	Novel	3.26	4.6	89.4	15.3	3.96	4.2	91.2	19.0	2.67	4.2	87.2	18.8	0.76	2.6	74.2	5.4
	Frequent	3.53	5.1	92.7	16.9	3.57	3.8	97.0	22.3	2.45	3.7	100.2	27.4	0.09	2.4	86.6	15.1

**Table 2:** Means and standard deviations for auditory N2 latencies (ms) and amplitudes (μV) at midlines by age-group (years).

		4–5 years (*N* = 40)				6–7 years (*N* = 82)				8–9 years (*N* = 46)				10–12 years (*N* = 10)			
		Amplitude		Latency		Amplitude		Latency		Amplitude		Latency		Amplitude		Latency	
		Mean	SD	Mean	SD	Mean	SD	Mean	SD	Mean	SD	Mean	SD	Mean	SD	Mean	SD
Fz	Infrequent	−10.32	6.4	239.4	25.0	−9.84	6.8	230.3	22.4	−7.78	5.6	227.7	25.9	−7.24	6.0	237.8	15.1
	Novel	−4.07	9.7	245.7	23.6	−1.49	9.3	236.8	22.8	−4.71	9.4	241.6	25.8	−0.65	5.9	239.0	24.4
	Frequent	−5.78	6.7	232.0	19.1	−8.83	6.2	238.3	16.0	−8.24	5.5	236.9	23.2	−8.80	4.0	243.6	15.8

FCz	Infrequent	−8.34	6.1	240.9	26.4	−8.30	6.6	229.8	22.3	−6.20	5.7	229.5	28.3	−5.29	6.4	233.2	16.9
	Novel	0.95	8.2	242.8	26.6	2.01	8.7	234.2	21.8	0.48	9.4	241.3	26.5	5.90	7.8	236.0	44.7
	Frequent	−6.24	6.1	234.5	18.1	−9.05	5.5	240.2	17.5	−7.54	5.6	240.6	20.9	−8.11	3.8	240.6	15.1

Cz	Infrequent	−7.25	5.6	242.8	29.9	−6.89	5.6	228.7	21.3	−5.58	4.2	231.4	28.5	−4.50	5.7	233.2	17.3
	Novel	1.13	8.3	247.8	25.5	2.87	7.5	237.5	24.0	0.43	8.1	245.1	27.6	7.44	4.8	234.6	26.7
	Frequent	−6.59	5.4	231.6	20.5	−8.90	4.7	240.7	16.9	−7.25	5.5	240.1	19.3	−7.26	3.9	237.8	18.6

Pz	Infrequent	−6.56	5.2	245.0	34.9	−6.19	5.6	231.7	26.0	−5.41	4.0	232.9	33.1	−4.98	5.1	238.2	22.9
	Novel	−5.97	6.5	240.9	28.9	−3.75	6.4	243.4	25.5	−4.95	6.0	246.7	25.1	−2.68	5.9	220.0	16.1
	Frequent	−5.40	4.4	232.7	24.1	−6.94	3.8	236.3	24.7	−5.84	4.3	238.8	27.1	−5.70	3.7	233.8	22.9

**Table 3:** Means and standard deviations for auditory P3a latencies (ms) and amplitudes (μV) at midlines by age-group (years).

		4–5 years (*N* = 40)				6–7 years (*N* = 82)				8–9 years (*N* = 46)				10–12 years (*N* = 10)			
		Amplitude		Latency		Amplitude		Latency		Amplitude		Latency		Amplitude		Latency	
		Mean	SD	Mean	SD	Mean	SD	Mean	SD	Mean	SD	Mean	SD	Mean	SD	Mean	SD
Fz	Infrequent	−4.05	6.0	321.5	35.6	−2.44	6.1	314.5	28.8	−0.31	7.0	320.1	31.3	−0.32	7.3	332.6	33.3
	Novel	3.18	9.8	318.0	30.2	7.38	9.3	319.1	31.6	6.81	9.4	329.0	31.4	12.76	5.8	337.2	24.1
	Frequent	2.58	6.4	320.6	24.5	−0.77	5.6	316.1	30.5	−1.17	6.0	317.4	37.6	−1.96	5.1	326.2	25.5

FCz	Infrequent	−0.92	6.7	331.5	32.1	0.16	6.0	314.4	27.2	1.54	6.9	316.7	28.8	2.61	6.4	312.8	35.1
	Novel	8.21	8.9	317.3	29.1	11.74	8.7	315.6	29.0	10.06	8.8	323.4	27.9	16.97	6.2	321.8	29.2
	Frequent	2.23	6.0	321.2	25.5	−0.84	5.3	322.1	29.9	−0.35	5.8	320.4	32.3	−2.34	4.4	332.6	25.9

Cz	Infrequent	−1.26	6.4	326.4	35.2	0.37	5.4	310.8	27.6	1.12	5.0	313.9	28.9	2.22	5.8	300.6	26.4
	Novel	6.15	8.9	311.2	29.5	10.33	7.9	309.5	30.0	7.55	7.8	325.8	30.1	15.70	5.0	323.0	23.7
	Frequent	1.07	6.2	321.1	28.4	−1.11	5.3	321.3	27.7	−0.18	5.4	324.0	33.5	−2.29	4.3	322.6	35.6

Pz	Infrequent	−1.77	5.5	331.5	39.1	0.38	4.7	314.7	41.0	0.82	5.0	317.2	33.5	1.10	2.9	318.8	36.6
	Novel	1.76	5.5	326.7	36.0	5.30	6.4	332.4	29.4	5.19	6.6	343.1	22.0	10.91	6.1	329.4	25.3
	Frequent	0.68	5.1	321.1	31.4	−0.78	4.0	316.8	35.6	0.51	3.9	329.2	37.0	−1.43	4.1	309.2	38.5

**Table 4:** Means and standard deviations for visual P3a and N170 latencies (ms) and amplitudes (μV) at midline and T5/T6 sites by age-group (years).

		4–5 years (*N* = 40)				6–7 years (*N* = 82)				8–9 years (*N* = 46)				10–12 years (*N* = 10)			
		Amplitude		Latency		Amplitude		Latency		Amplitude		Latency		Amplitude		Latency	
		Mean	SD	Mean	SD	Mean	SD	Mean	SD	Mean	SD	Mean	SD	Mean	SD	Mean	SD
Fz	Infrequent	0.77	10.1	406.7	50.1	6.27	10.3	415.9	36.3	5.84	9.4	401.6	42.7	6.18	7.3	425.8	47.3
	Novel	−4.58	8.3	386.4	50.0	−0.60	8.5	392.1	40.0	−3.93	8.1	378.2	37.8	−1.13	6.1	404.0	51.2
	Frequent	−0.88	5.2	410.0	51.2	3.14	7.3	410.2	45.5	1.01	5.2	395.7	46.5	2.04	4.6	406.8	46.1

FCz	Infrequent	0.48	8.4	415.6	49.5	6.81	9.8	413.6	37.3	5.60	8.0	404.8	46.6	6.95	7.4	427.2	49.4
	Novel	−3.94	8.2	395.6	50.1	0.62	8.2	393.5	44.4	−2.81	8.1	378.0	39.9	1.69	7.2	410.0	52.5
	Frequent	0.16	5.1	414.9	53.5	3.58	7.1	411.0	46.7	1.32	4.8	391.3	44.0	3.30	4.8	415.0	53.1

Cz	Infrequent	0.47	7.0	415.1	49.2	7.42	9.3	413.9	52.8	5.60	7.7	407.0	54.4	5.90	7.5	424.2	56.2
	Novel	−3.71	8.9	407.2	53.9	0.71	7.7	398.2	47.8	−1.71	8.8	399.5	52.9	1.51	8.1	406.2	57.7
	Frequent	−0.34	4.3	414.0	51.2	4.11	6.9	410.3	51.7	1.87	4.3	403.9	54.5	3.35	4.8	437.4	49.4

Pz	Infrequent	3.76	6.3	417.7	49.1	10.55	8.7	413.1	54.8	8.53	7.5	408.5	58.1	8.66	5.1	446.2	54.5
	Novel	4.07	9.6	410.2	45.7	7.77	9.2	410.9	55.0	5.62	10.5	426.3	56.2	8.87	6.4	432.8	61.8
	Frequent	3.96	5.6	410.2	51.7	8.71	7.2	414.7	59.2	6.20	6.9	412.1	56.4	7.36	5.2	424.2	56.9

T5	Infrequent	(Measures not available)^a^				−4.82	5.4	219.7	23.3	−2.32	6.0	220.7	28.3	−1.95	5.3	209.6	25.3
	Novel					−8.94	5.8	230.6	21.9	−7.49	5.3	224.1	25.2	−6.61	3.9	230.4	23.8
	Frequent					−9.36	7.5	229.3	24.2	−6.96	5.1	219.3	25.9	−6.21	4.3	218.6	30.1

T6	Infrequent	(Measures not available)^a^				−4.12	5.7	224.6	20.5	−2.37	5.9	220.9	23.4	−1.20	6.2	202.6	29.9
	Novel					−10.03	6.0	237.0	18.9	−8.35	5.4	226.5	26.0	−7.53	6.4	218.0	24.8
	Frequent					−11.33	7.3	232.2	22.6	−8.89	5.6	223.5	26.3	−8.87	8.8	203.0	21.3

a The measures for T5 and T6 are unavailable for younger children as we used a different montage.

**Table 5:** Means and standard deviations for visual Nc component amplitudes at midline sites for each age-group.

		4–5 years		6–7 years		8–9 years		10–12 years	
		Amplitude		Amplitude		Amplitude		Amplitude	
		Mean	SD	Mean	SD	Mean	SD	Mean	SD
Fz	Novel	−3.72	8.7	1.50	7.7	0.83	6.9	1.19	5.4
	Frequent	−11.01	8.6	−6.56	8.3	−9.75	7.7	−5.89	6.3
	Infrequent	−4.46	4.4	−0.39	5.4	−1.65	4.4	0.17	3.2

FCz	Novel	−2.25	6.3	2.90	7.7	1.73	6.2	2.85	5.3
	Frequent	−7.81	8.9	−3.44	7.8	−6.34	6.9	−3.09	6.4
	Infrequent	−2.80	4.7	0.55	5.3	−0.93	4.0	0.80	3.1

Cz	Novel	0.40	6.4	5.30	7.6	3.74	5.7	4.15	5.5
	Frequent	−5.56	10.0	−1.23	7.9	−2.47	6.8	−0.59	8.2
	Infrequent	−1.12	4.4	2.29	5.5	1.08	3.7	2.37	3.5

Pz	Novel	5.87	7.7	8.67	8.4	7.73	6.1	7.46	6.2
	Frequent	2.66	8.0	5.90	8.9	5.17	8.0	7.56	8.6
	Infrequent	3.84	5.0	5.73	5.8	4.73	4.3	5.60	4.4
